# Connecting materials sciences with fungal biology: a sea of possibilities

**DOI:** 10.1186/s40694-022-00137-8

**Published:** 2022-03-01

**Authors:** Vera Meyer

**Affiliations:** Chair of Applied and Molecular Microbiology, Technische Universität Berlin, Straße des 17. Juni 135, 10623 Berlin, Germany

## Abstract

The Special Issue “Connecting materials science with fungal biology” celebrates recent breakthroughs in the fabrication of fungal-based materials, all of which have been made possible by the interdisciplinary and transdisciplinary collaboration of fungal biologists and biotechnologists with artists, designers, materials scientists, and architects. It features conceptual considerations and latest developments of these joint research efforts and the paradigm shift that is involved. The aim of this collection of twelve papers is to highlight the infinite possibilities for the development of innovative fungal-based materials which can be realized through integrating the knowledge and methods from different disciplines.

## Main text

How we want to live in the future is one of the central questions that concern us all, whether as citizens or scientists. The importance of respecting planetary boundaries and the necessary transition from a petroleum-based, linear towards an environmentally sustainable, circular economy are beyond dispute. Importantly, fungal biology and biotechnology can contribute significantly to these transformation efforts by researching and developing new classes of fungal-based materials that have the potential to replace many petroleum-derived materials. Hereby, residual streams from renewable agricultural and forestry biomass become biotechnologically transformed into fungal biomass to generate biomaterials with infinite application possibilities. These are mind-blowing and range from (i) fungal composite materials used as insulating and building materials, (ii) pure mycelium materials harnessed as leather-like materials, textiles, self-repairing electronic skins, or filters for air and water purification, (iii) chitin- and glucan-based nanomaterials derived from fungal mycelium for health care, cosmetics and veterinary ingredients, to (iv) fungal-engineered wood for high-quality tone wood or controlled spalting, to name but a few [1].

Research into and the development of fungal-based materials is an interdisciplinary effort that is not trivial at all. It aims at a holistic understanding and rational optimization of all biological and technological aspects that impact the final material properties. The mechanical, physical, chemical, thermal, constructional, sensual, aesthetic, or architectural characteristics are clearly dependent on the genetic make-up of the fungal production organism used (e.g. its growth profile, branching pattern, hydrophobicity, cell wall architecture, and enzymatic equipment), the properties of the agricultural and forestry substrates (e.g. shape, size, texture, and molecular composition), and the manufacturing and processing procedures (e.g. substrate pretreatment, cultivation conditions, and post-treatment, such as drying, pressing, or coating). Only fundamental and, thus, comprehensive understanding of all these interacting, multiscale influencing parameters will lay the scientific foundations for defined manufacturing processes and reproducible property profiles of fungal-based materials and eventually break new ground in materials science with these ‘living materials.’

The application of fungal-based materials is a tangible and, therefore, realistic future scenario and—if successfully implemented into our daily lives—could contribute significantly to the transformation of a petroleum-based economy into a bioeconomy, thus, making an active contribution to combat climate change. The vision that in the not-so-distant future we will be living in houses co-designed with fungi, using furniture grown with the help of fungi, and even being able to wrap ourselves in fungal clothing [1] could change the way we live and work decisively and prove disruptive, even threatening, to existing markets. It is, therefore, important that these research efforts must be accompanied by an intensive dialog with society. The core questions are how fungal-based materials and their application scenarios can be proactively discussed with society, and what paths must be taken to anchor them sustainably in society. I am convinced that various scientific communication formats and active participation structures for citizens, artists, and designers are, thus, of utmost importance in order to jointly discuss scenarios for a future life and living with and through fungi. Methods from social sciences, such as participation and acceptance research, have to be integrated in fungal materials research to explore the new materials together with society. These transdisciplinary collaborations will help us to understand how the topic of novel fungal-based materials can be introduced to the nonscientific public and what public spaces and formats need to be created so that civil society becomes an active part of the transformation process.

Given the importance of this still young research field of ‘living materials’ in driving new science and contributing to a sustainable future, the journal *Fungal Biology and Biotechnology* is happy to feature twelve invited articles in the Special Issue “Connecting materials science with fungal biology.” This is the first Special Issue ever in the field of fungal-based materials and covers articles that have been published between Spring 2021 and Spring 2022 in the subfields (i) composite materials, (ii) pure mycelium materials, (iii) nanomaterials, and (iv) fungal-engineered wood. It also includes articles addressing the importance of communication within and outside the community and of transdisciplinary research.

The research articles by Elsacker et al. [2], Chen et al. [3] and Pohl et al. [4] discuss the potentials of the white-rot fungi *Trametes versicolor* and *Fomes fomentarius* for the fabrication of fungal composite materials and highlight possibilities for their improvement and even use in 3D printing (additive manufacturing) when biopolymers, such as alginates or cellulose, are added as binders. Vandelook et al. [5] contributed with a primer which features the endless application possibilities of pure fungal mycelium from *Ganoderma*, *Fomes*, *Trametes*, *Pycnoporus*, or *Perenniporia* spp. as leather alternatives, textiles, or foams and summarizes the main companies driving the development of such new consumer products. Adamatzki et al. [6] open the door in their research article to the development of biocomputers through the application of living fungal mycelium from *Ganoderma resinaceum* as an intelligent skin that reacts to mechanical and optical stimuli. Van Wylick et al. [7] take up another perhaps surprising application potential of fungal mycelium and discuss in their review the usage of the living fungal mycelium of alkali-tolerant species from the genera *Aspergillus*, *Fusarium*, or *Trichoderma* to heal concrete cracks by the precipitation of calcium carbonate through biomineralization. Cord-Landwehr and Moerschbacher [8] call attention in their primer to the fungal polymers chitin and chitosans, which can be harnessed as nanomaterials in medical, pharmaceutical, cosmetics, paper, plastics, and textile applications.

**Fig. 1:**
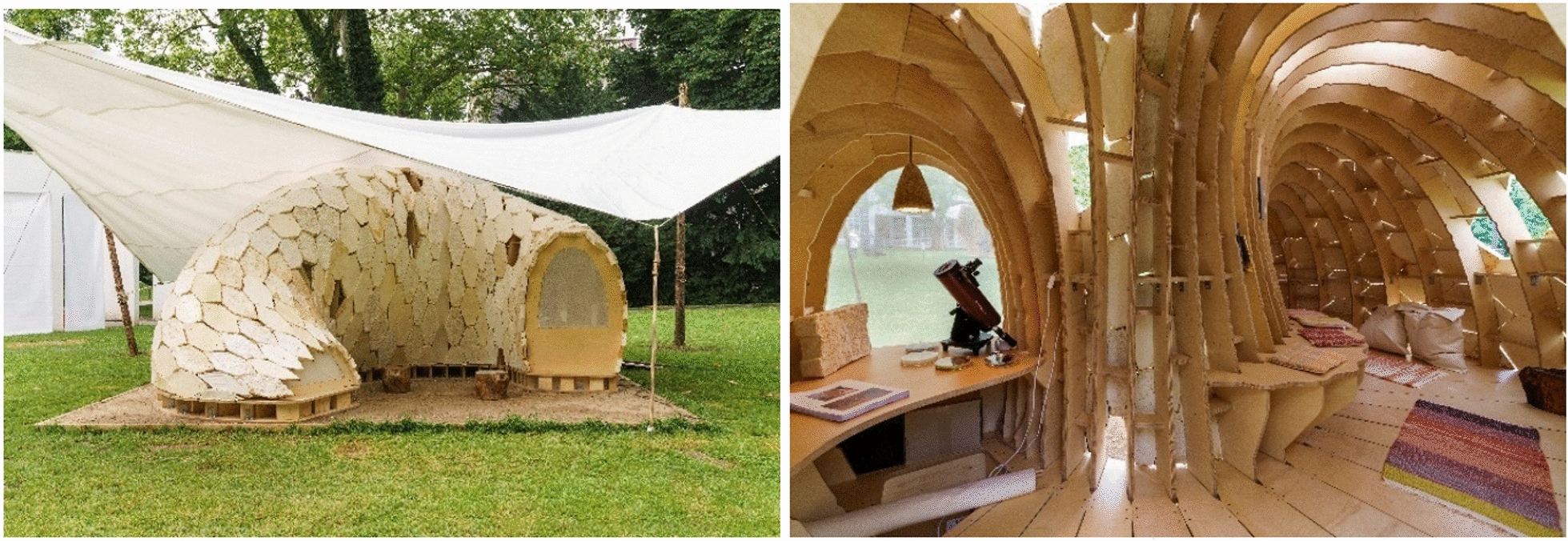
Exterior (left) and interior (right) view of MY-CO SPACE. The pavilion was designed for the tinyBE #1 exhibition in Frankfurt am Main (Germany) and open to the public as well as bookable for overnight stays in summer 2021 [12]. MY-CO SPACE draws attention to the deconstruction and reconstruction potential of fungi and how they transform and recycle organic material. It invites one to a journey into the microscopically small but macroscopically experienceable world of fungi. The needs of the inhabitants are secured by fungi—they live sheltered in a fungal pavilion, sit on fungal furniture, use woven textiles stained with fungal pigments, and are illuminated by fungal lamps at night. A microscope assists in the observation of fungal mycelia formation, and a telescope offers contact to the outside world and the macrocosm. © MY-CO-X, image credits: Wolfgang Günzel, tinyBE

Almpani-Lekka et al. [9] review six artistic-architectural works that highlight the paradigm shift that fungal-based materials could offer to a circular and sustainable architecture of the future in which the construction of new buildings could be potentially carbon-negative. The artistic and design concept, for example, of MY-CO SPACE (Fig. 1), designed by the Berlin-based interdisciplinary SciArt collective MY-CO-X, is among the artworks spotlighted. This pavilion was inspired by manned spacecraft programs of the last century and transported their central design question “How can physical-technical structures and essential living functions be integrated in the smallest space in such a way that people can live and work under conditions of weightlessness and extreme physical stress?” [10] to today’s challenges: “How can biological-technical structures and essential living functions be integrated in the smallest possible space in such a way that people can still live and work light-hearted under conditions of limited resources?” [11].

Van den Brandhof and Wösten initiate [13] the very important discussion on risk assessment in their review to understand whether fungal-based materials are truly sustainable regarding human and environmental health. Questions that need to be discussed and researched by the community should not only cover the manifold opportunities of new fungal-based materials but also assess the proposed fungal production strains, i.e. whether they are potentially pathogenic, may form mycotoxins, attract insects though volatile compounds they produce, or even become invasive species. These and other major issues that need to be discussed within the community (e.g. on how to standardize manufacturing processes and, thus, material properties or how to integrate life cycle assessments to determine environmental footprints) have been summarized by Attias et al. [14]. They refer in their meeting report to the first international meeting dedicated fully to fungal-based materials. This “Fungal Mycelium Materials Mini Meeting” was held remotely in August 2021 and brought together delegates from ten European research institutions with scientific backgrounds in materials science, fungal bio(techno)logy, engineering, art, and architecture [14]. This diversity in educational backgrounds was and is an opportunity and a challenge at the same time. Cross-disciplinary communication and collaboration is not easy as different languages, terminologies, perspectives, and even ways of thinking must merge before a fruitful dialog that benefits all can start. This challenge is intensified if, in addition, communication with society is strived for. The research article by Sharma and Meyer [15], therefore, discusses the chances of artist-in-residence programs to bridge the divide between science, art, and society. A video artwork [16] and the Living Color Database (www.color.bio) were developed around the central theme of microbial pigmentation as part of the 2021 “Colors of Life” residency of the artist and designer Sunanda Sharma, that aimed to provide paths for citizens, artists, designers, and scientists to understand microbial life through the lens of color.

Transformation processes need time to evolve. Even the steam engine did not suddenly appear but was developed over many decades before it became fully operational and worked efficiently. It took many years for it to be established and developed into a new viable economic sector that others followed, and even more years before society was caught up in the industrial revolution. The beginnings of transformation processes are, thus, often forgotten. Therefore, the last article of this Special Issue is dedicated to the beginnings of fungal-based materials research and—unexpectedly—reverts to the 1940s–1950s. This is when the pioneering work of the engineer Walther Luthardt and his invention of the so-called *Myko-Holz* (Myco-wood) started in Thuringia (Germany). As summarized in the historic review by Stange and Wagenführ [17], he worked with *Pleurotus ostreatus* and *Trametes versicolor*, laid the technical foundation for controlled fungal decomposition of wood with his investigations and patents, and developed protocols for mushroom spawn (inoculation paste) production based on pure fungal cultures and wood sawdust. The true beginnings of fungal composite materials research, therefore, go back to the middle of the last century.

It is hoped that this collection of twelve papers will provide an excellent entry point for not only interested readers who are new to the field but also the experts of different disciplines to help them understand the current state of the art in fungal materials research, the knowledge gaps, and areas ripe for development. There is still much to learn, and more joint and cross-disciplinary research is needed to harness the endless opportunities that fungal materials might offer. In my opinion, this potential can only be tapped through the triad of science, art, and society.

## Data Availability

All links to external webpages provided in the text were consulted in February 2022.

## References

[1] Meyer V, Basenko EY, Benz JP, Braus GH, Caddick MX, Csukai M (2020). Growing a circular economy with fungal biotechnology: a white paper. Fungal Biol Biotechnol.

[2] Elsacker E, Vandelook S, Damsin B, Van Wylick A, Peeters E, De Laet L (2021). Mechanical characteristics of bacterial cellulose-reinforced mycelium composite materials. Fungal Biol Biotechnol.

[3] Chen H, Abdullayev A, Bekheet MF, Schmidt B, Regler I, Pohl C (2021). Extrusion-based additive manufacturing of fungal-based composite materials using the tinder fungus *Fomes fomentarius*. Fungal Biol Biotechnol.

[4] Pohl C, Schmidt B, Nunez Guitar T, Klemm S, Gusovius HG, Platzk S (2022). Establishment of the basidiomycete *Fomes fomentarius* for the production of composite materials. Fungal Biol Biotechnol.

[5] Vandelook S, Elsacker E, Van Wylick A, De Laet L, Peeters E (2021). Current state and future prospects of pure mycelium materials. Fungal Biol Biotechnol.

[6] Adamatzky A, Gandia A, Chiolerio A (2021). Towards fungal sensing skin. Fungal Biol Biotechnol.

[7] Van Wylick A, Monclaro AV, Elsacker E, Vandelook S, Rahier H, De Laet L (2021). A review on the potential of filamentous fungi for microbial self-healing of concrete. Fungal Biol Biotechnol.

[8] Cord-Landwehr S, Moerschbacher BM (2021). Deciphering the ChitoCode: fungal chitins and chitosans as functional biopolymers. Fungal Biol Biotechnol.

[9] Almpani-Lekka D, Pfeiffer S, Schmidts C, Seo S-I (2021). A review on architecture with fungal biomaterials: the desired and the feasible. Fungal Biol Biotechnol..

[10] Meuser P. Galina Balaschowa. Architektin des sowjetischen Raumfahrtprogramms. Berlin: DOM publishers; 2014. ISBN 978-3-86922-345-2.

[11] MY-CO-X. 2021. https://tinybe.org/en/artists/my-co-x/. Accessed 16 Feb 2022.

[12] tinyBEjournal. MY-CO-X. 2021. https://tinybe.org/en/. Accessed 16 Feb 2022.

[13] Van den Brandhof JG, Wösten H. Risk assessment of fungal materials. Fungal Biol Biotechnol. 2022. (in press). 10.1186/s40694-022-00134-x PMC8876125 35209958

[14] Attias N, Livne A, Abitbol T (2021). State of the art, recent advances, and challenges in the field of fungal mycelium materials: a snapshot of the 2021 Mini Meeting. Fungal Biol Biotechnol.

[15] Sharma S, Meyer V (2022). The colors of life: an interdisciplinary artist-in-residence project to research fungal pigments as a gateway to empathy and understanding of microbial life. Fungal Biol Biotechnol.

[16] Sharma S, Meyer V, Colors of Life I. A film. 2021. https://www.youtube.com/watch?v=rygnsEiIrgw. Accessed 16 Feb 2022.

[17] Stange S, Wagenführ A. 70 years of wood modification with fungi. Fungal Biol Biotechnol. 2022. (in press). 10.1186/s40694-022-00136-9 PMC8931968 35303960

